# Molecular cloning, expression and biochemical characterisation of a cold-adapted novel recombinant chitinase from *Glaciozyma antarctica *PI12

**DOI:** 10.1186/1475-2859-10-94

**Published:** 2011-11-04

**Authors:** Aizi Nor Mazila Ramli, Nor Muhammad Mahadi, Amir Rabu, Abdul Munir Abdul Murad, Farah Diba Abu Bakar, Rosli Md Illias

**Affiliations:** 1 Department of Bioprocess Engineering, Faculty of Chemical Engineering, Universiti Teknologi Malaysia, 81310 Skudai, Johor, Malaysia; 2 Malaysia Genome Institute (MGI), Jalan Bangi Lama, 43000 Kajang, Selangor, Malaysia; 3 School of Biosciences and Biotechnology, Faculty of Science and Technology, Universiti Kebangsaan Malaysia, 43600 Bangi, Selangor, Malaysia

**Keywords:** Cold-adapted chitinase, ***Glaciozyma antarctica ***, **PI12. Psychrophilic yeast**, *Pichia pastoris*

## Abstract

**Background:**

Cold-adapted enzymes are proteins produced by psychrophilic organisms that display a high catalytic efficiency at extremely low temperatures. Chitin consists of the insoluble homopolysaccharide β-(1, 4)-linked *N*-acetylglucosamine, which is the second most abundant biopolymer found in nature. Chitinases (EC 3.2.1.14) play an important role in chitin recycling in nature. Biodegradation of chitin by the action of cold-adapted chitinases offers significant advantages in industrial applications such as the treatment of chitin-rich waste at low temperatures, the biocontrol of phytopathogens in cold environments and the biocontrol of microbial spoilage of refrigerated food.

**Results:**

A gene encoding a cold-adapted chitinase (CHI II) from *Glaciozyma antarctica *PI12 was isolated using Rapid Amplification of cDNA Ends (RACE) and RT-PCR techniques. The isolated gene was successfully expressed in the *Pichia pastoris *expression system. Analysis of the nucleotide sequence revealed the presence of an open reading frame of 1,215 bp, which encodes a 404 amino acid protein. The recombinant chitinase was secreted into the medium when induced with 1% methanol in BMMY medium at 25°C. The purified recombinant chitinase exhibited two bands, corresponding to the non-glycosylated and glycosylated proteins, by SDS-PAGE with molecular masses of approximately 39 and 50 kDa, respectively. The enzyme displayed an acidic pH characteristic with an optimum pH at 4.0 and an optimum temperature at 15°C. The enzyme was stable between pH 3.0-4.5 and was able to retain its activity from 5 to 25°C. The presence of K^+^, Mn^2+ ^and Co^2+ ^ions increased the enzyme activity up to 20%. Analysis of the insoluble substrates showed that the purified recombinant chitinase had a strong affinity towards colloidal chitin and little effect on glycol chitosan. CHI II recombinant chitinase exhibited higher V_max _and K_cat _values toward colloidal chitin than other substrates at low temperatures.

**Conclusion:**

By taking advantage of its high activity at low temperatures and its acidic pH optimum, this recombinant chitinase will be valuable in various biotechnological applications under low temperature and acidic pH conditions.

## Background

Extremophiles are microorganisms that can grow and thrive in extreme environments. Proteins, especially enzymes, isolated from the extremophiles are of particular interest because of their ability to function effectively and remain stable near extreme conditions [1]. Psychrophiles are organisms that live at very low temperatures and can be found in several perennially cold environments, such as the Antarctic. The survival of the psychrophilic organisms at low temperatures (cold-adaptation) are due to several factors such as temperature sensing, reduced membrane fluidity, stabilised inhibitory nucleic acid structures, the formation of intracellular crystalline ice and cellular responses that counteract solute uptake rates and lowered enzyme reactions [2]. Recently, psychrophilic enzymes are becoming more attractive in industrial applications, partly because of ongoing efforts to decrease energy consumption. At low temperatures, the kinetic energy of reacting molecules is too low to allow reactions to occur. Psychrophilic or cold-adapted enzymes compensate in this situation by having a highly flexible protein structure and conformation, thereby increasing their thermolability and a high catalytic efficiency at a low energy cost [2,3]. To date, many cold-adapted enzymes have been successfully isolated and their expression studies have also been conducted. This includes the glycosyl hydrolase group of enzymes, such as lipases [4], α-Amylases [5] and chitinases [6,7].

Chitin is the most abundant biopolymer found in nature after cellulose and attracted special interest as a reusable material [6,8]. This polysaccharide is a crucial structural component in fungal cell walls and certain green algae and is a major constituent in the shells, cuticles and exoskeletons of worms, molluscs and arthropods, including crustaceans and insects [7]. Chitin comprises 20 to 58% of the dry weight of the marine invertebrates, which include shrimp, crabs, squids, oysters and cuttlefish [9]. The enormous amounts of chitin continuously generated in nature require disposal and recycling on a formidable scale [10]. Previous studies revealed the broad range applications of chitin in various biochemical, food and chemical industries. Patil *et al*. [11] showed that chitin can be used in human health care as an antimicrobial, anticholesterol or antitumor agent. Chitin and its derivatives are also used in wastewater treatment, drug delivery, wound healing and dietary fibre [12]. Due to chitin's important biological role, its synthesis and degradation has been the subject of extensive research.

Chitinases catalyse the hydrolysis of β-1, 4-linkages in chitin. Many organisms produce chitinases for different purposes [13]. Chitinases produced by bacteria and plants are important for nutritive purposes and in fungal invasion, respectively. All chitin-containing organisms such as fungi and yeast produce chitinases (EC 2.4.1.14) and chitin synthase (EC 2.4.1.16) to mediate cell wall synthesis and growth [12,14]. Chitinases are classified into two families of glycosyl hydrolases, family 18 and family 19, based on the amino acid sequence of the catalytic regions [15]. Family 18 contains chitinases from bacteria, fungi, viruses, animals and some plants [7]. Cold-adapted chitinases are always characterised by low optimal temperatures and increased structural flexibility that is achieved through a combination of structural features [16]. Heat labile and cold-adapted chitinases have been reported from several psychrophilic bacteria [7,17], plants [18] and fungi [19]. However, a cold-adapted chitinase from psychrophilic or psychrotolerant yeast has yet to be reported.

Due to difficulties in getting significant amount of native chitinase of *G. antarctica *PI12 for protein purification, initial expression of the CHI II gene was carried out in *E. coli *system. However no expression was observed (data not shown). This could be due to the reduced stability of recombinant psychrophilic proteins expressed in a mesophilic host such as *E. coli *[20,21]. An alternative host to *E. coli *is the methylotrophic yeast, *P. pastoris *which can be regarded as a moderate psychrotrophic organism that can grow at temperature as low as 12°C. This strain has emerged as a powerful and inexpensive expression system for the production of the eukaryotic recombinant proteins [22].

In this study, we described the isolation and recombinant expression of a psychrophilic chitinase (CHI II) gene from *G. antarctica *PI12 in *P. pastoris*. Purification and characterisation of the expressed recombinant CHI II were also conducted. Subsequent biochemical characterisation of this enzyme suggests its usefulness in some biotechnology applications.

## Results and Discussion

### Cloning and sequence analysis of CHI II from G. antarctica PI12

A nucleotide sequence obtained from a GSS survey of the ***G. antarctica *PI12** genome was identified to encode the consensus domain of the glycosyl hydrolase family 18 using NCBI databases. Further analyses suggest that the nucleotide sequence encoded part of a chitinase gene sequence. Isolation of the full-length chitinase gene was accomplished by RT-PCR followed by RACE amplification. A DNA fragment of approximately 500 bp was obtained from the RT-PCR method. Due to a lack of DNA information for the *G. antarctica *PI12 chitinase, RACE techniques were used to amplify the full-length cDNA sequence. The RACE method was conducted using the RNA of *G. antarctica *PI12 and the 5' and 3' portion sequence of the full-length cDNA was obtained by 5' and 3' RACE, respectively. About 1100 bp was amplified from 5' RACE and approximately 1200 bp from 3' RACE. Conjugation of the 5' and 3' fragments revealed a full-length chitinase cDNA of 1215 bp containing a 404 bp ORF, a 448 bp 5' untranslated region and a 121 bp untranslated flank at 3' end. The latter includes a polyadenylation signal AATAAA located 23 bp to the 5' side of the poly A tail and a ATTTA sequence, which is involved in the targeting of mRNAs for rapid turnover. Based upon the RACE sequences, a set of primers was designed (CHI-For and CHI-Rev) and the full-length cDNA sequence was amplified via the RT-PCR method. The cDNA sequence was deposited into GenBank with the Accession No.: JF901326.

The DNA sequence encodes a 404 amino acid protein with a calculated molecular weight of 42.9 kDa and a theoretical pI of 9.65. Previous studies reported that the chitinases from various organisms had a molecular mass of about 40-80 kDa. The recombinant chitinase from *Clonostachys rosea *had a molecular mass of 43.8 kDa [23], whereas the recombinant chitinase from *Chaetomium cupreum *and *Vibrio *sp. Fi:7 was found to have a molecular mass of 58 kDa [24] and 79.4 kDa [25], respectively. A SignalP [26] prediction revealed that the CHI II protein contains a putative N-terminal signal peptide of 19 amino acids in length with a predicted cleavage site located between A19 and E18 (THA|||EL). This result suggested that this enzyme is secretory in nature. Analysis of the CHI II sequence by NetNGlyc 1.0 Server [27] showed that there is one potential N-linked glycosylation site at position -371. Interproscan [28] analysis predicted that CHI II encodes a glycosyl hydrolase family 18 member because of the amino acid segment "FDGVDLDWE" at nucleotide position 498, which matches the consensus catalytic sequence pattern [LIVMFY]-[DN]-G-[LIVMF]-[DN]-[LIVMF]-[DN]-x-E of chitinases in family 18 glycosyl hydrolases. The CHI II InterPro Accession No. is IPR001223, indicating that it is a novel member of the family 18 glycosyl hydrolases (endochitinase) and belongs to the chitinase class II.

**Figure 1:**
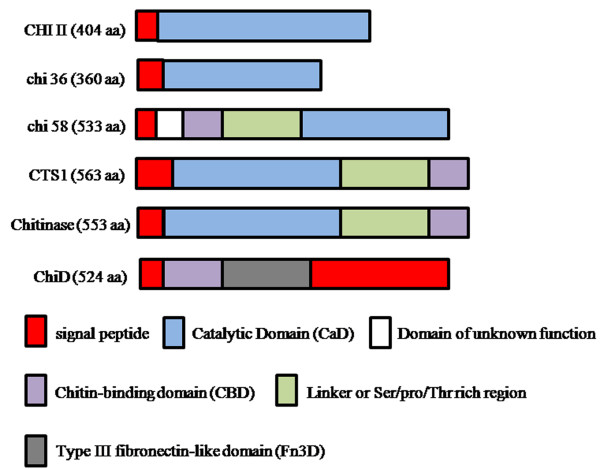
**Domain organisation of family 18 chitinases; *G. antarctica *PI12 (CHI II), *Bacillus cereus *(chi 36) **[29], ***Chaetomium cupreum *(chi 58) **[24], ***Saccharomyces cerevisiae *(CTSI) **[56], ***Lacanobia oleracea *(Chitinase) **[57]**and *Bacillus circulans *WL-12 (ChiD) **[58].

Based on a Blast search analysis, CHI II showed low similarity to all proteins in the database with an identity of 34% to chitinase from *Puccinia triticina *and 33% to endochitinase from *Amanita muscaria*. A low identity of the CHI II sequence with the available sequences in the database suggested that this chitinase from *G. antarctica *PI12 was a newly isolated chitinase sequence from yeast. CHI II appears to be a simple and compact chitinase with an N-terminal sequence encoding for a signal peptide and a C-terminal catalytic domain (CaD). Interestingly, neither the chitin-binding domain (CBD) nor the Ser/Pro/Thr rich linker, which were often associated with the catalytic domain in family 18 chitinases, was found in CHI II (Figure 1). This finding supports the study by Wang *et al*. [29] that showed that a single CaD is sufficient for the catalytic activity of chitinase and further suggested that the CBD may facilitate hydrolysis of an insoluble substrate [30] but was not required for chitinase activity. Figure 1 shows the domain organisations of family 18 chitinases that demonstrates the similarity of the CHI II domain structure with the chitinase from *Bacillus cereus *(chi36) [29] and the differences from others. It is unclear whether CHI II from *G. antarctica *PI12 evolved from multidomain chitinases as an alternative mechanism to proteolytic cleavage for the acquisition of high efficiency soluble chitinolytic activity.

**Figure 2:**
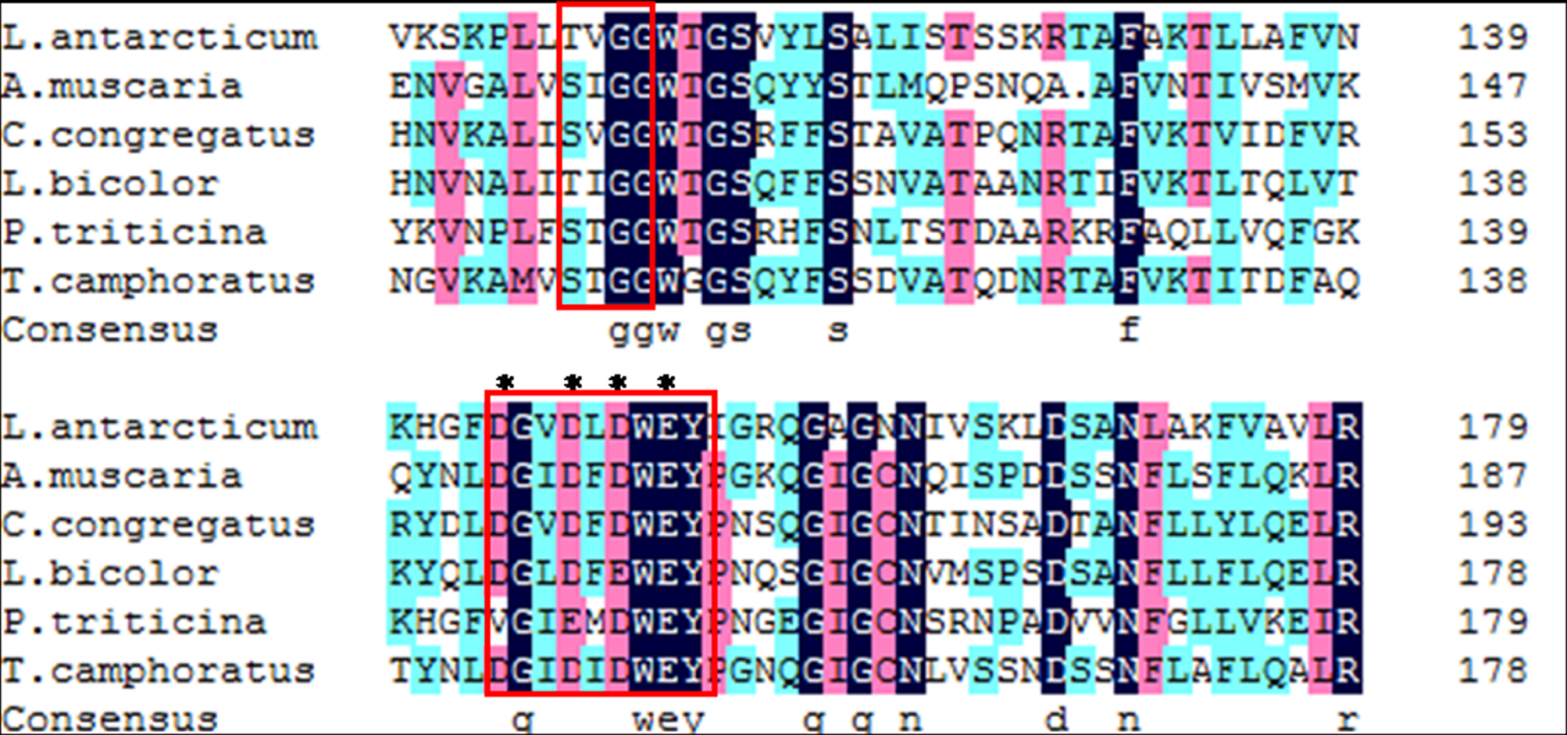
**Multiple sequence alignment of the core region of the catalytic domains between CHI II and some eukaryotic chitinases**. Highly conserved regions, SxGG and DxxDxDxE, are boxed. Residues responsible for the catalytic activity are indicated with asterisks. Sequence fragments displaying the typical motifs with the *G. antarctica *PI12 catalytic domain include *Amanita muscaria, Laccaria bicolor, Coprinellus congregatus, Puccinia triticina *and *Taiwanofungus camphorates*.

A multiple sequence alignment of the catalytic domain was conducted using the DNAMAN programme (Figure 2). Five catalytic domains of chitinases from other eukaryotes were chosen and aligned based on their strong identity to the chitinase from *G. antarctica *PI12. This core region contains two conserved amino acid regions, each consisting of SxGG and DxxDxDxE, which are highly conserved among the family 18 chitinases and may constitute the catalytic pocket [15,23]. Importantly, residues that are essential for chitinase activity, particularly Asp144, Asp147, Asp149 and Glu151, were also observed in the CHI II catalytic domain, implying the crucial role of these residues in the catalytic activity and structure of chitinases [31].

### Multiple sequence alignment and phylogenetic analysis

To investigate the evolutionary relationship among the cold-adapted chitinase identified in this work and others reported in the literature, phylogenetic analysis was performed. The search for complete protein sequence was explored using the NCBI BlastP service. A total of 24 chitinase sequences (all hits with an e-value lower than 6 × 10^-20^) were downloaded and aligned using clustal X. From this alignment, a NJ tree was constructed to examine the distances among these sequences. An NJ tree was then inferred and the tree topology was analysed using bootstrapping (1000 replicates). The chitinase of *Streptomyces griseus*, which did not coincide with the taxonomic status of the CHI II, was used as an out-group in order to root the tree.

**Figure 3:**
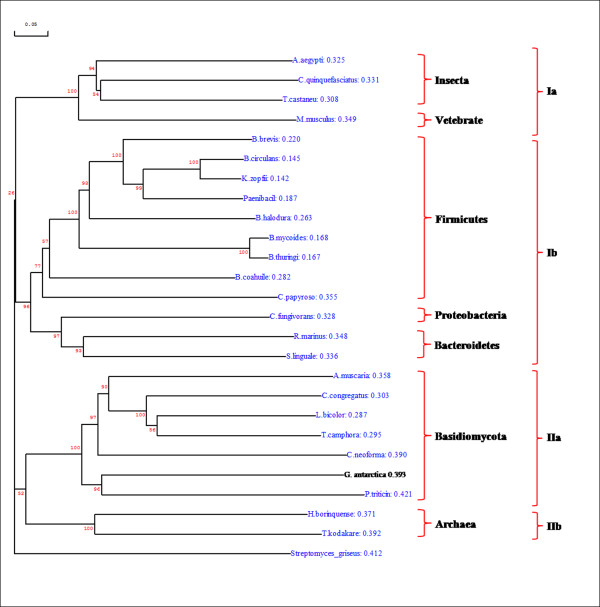
**Phylogenetic tree showing the relationship between CHI II from *G. antarctica *PI12 and other chitinases**. The chitinase gene of *S. griseus *was used as an out-group to root the tree. Confidence values were assessed from 1,000 bootstrap replicates of the original sequence data.

The analysis summarised in Figure 3 shows that the chitinase sequences clustered into two supported subgroups corresponding to clade I and II, which are both monophyletic clades. The monophyletic lineages provided a support for the hypothesis that clade I and II chitinases are likely to have evolved from one common ancestor [32]. Clade I consists of two subclades; chitinases from Vertebrate and Insecta were clustered and formed the subclade Ia, while chitinases from the bacteria domain (Firmicutes, Proteobacteria and Bacteroidetes) were clustered and formed the subclade Ib. Chitinases from Basidiomycota and Archaea were clustered and formed the second monophyletic clade (clade II) that consisted of subclade IIa (Basidiomycota) and subclade IIb (Archaea). CHI II chitinase from *G. antarctica *PI12 was clustered in the Basidiomycota group, which was well supported by bootstrapping (NJ: 100%). Homology values ranging between 21 to 54% were found when comparing CHI II with the other Basidiomycota chitinases. Orikoshi *et al*. [17] found that the redundancy of the chitinase genes within the same species may reflect their functional difference between related proteins and also the adaptive evolution of the chitinases. Bootstrap values, an index of the accuracy of the phylogenetic tree, were found to be higher outside the tree but lower inside the tree, which may indicate the low relative diversity of all the sequences used in the analysis [33].

### Heterologous expression of the recombinant CHI II in *P. pastoris*

**Figure 4:**
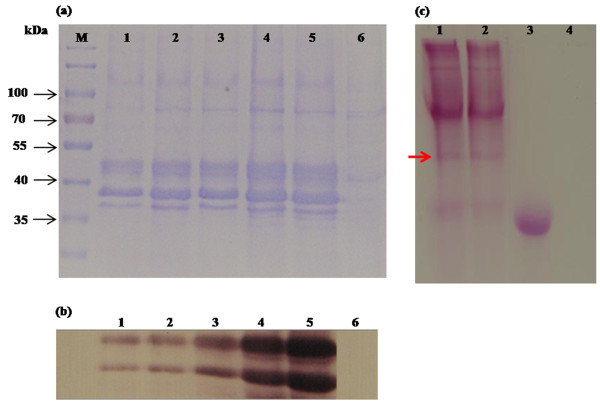
**SDS-PAGE, western blot and glycosylation analysis of recombinant CHI II protein in *P. pastoris *GS115**. (a) SDS-PAGE of transformant, M: protein molecular mass marker; Lanes 1-5: transformants induced by methanol for 1 to 5 days; Lane 6: control, GS115 transformed with empty pPICZαA and induced by methanol for 5 days. (b) Western blot of transformant, Lanes 1-5: transformants induced by methanol for 1 to 5 days; Lane 6: control, GS115 transformed with empty pPICZαA and induced by methanol for 5 days. (c) Glycosylation analysis of transformant, Lanes 1-2: transformants induced by methanol for 4 to 5 days; Lane 3: Horseradish Peroxidase Positive Control; Lane 4: Soybean Trypsin Inhibitor Negative Control. A 50 kDa protein was positively stained and indicated by an arrow.

The expression of CHI II was conducted in a *P. pastoris *expression system. Culture condition is one of the critical parameters that significantly affect cell growth and the yield of recombinant product. Stress imposed by cultivation conditions or strategies can lead to a decrease in cell viability, which in turn lowers productivity and induces cell lysis [34]. In this study, the highest extracellular chitinase activity was found to be 1.24 U ml^-1 ^at 120 h post-induction when cells were grown in BMMY medium (pH 6.0) at 25°C with 1% (v/v) methanol as inducer. Yield of the recombinant CHI II expressed in *P. pastoris *was shown to be significantly improved at low temperatures. This could be due to the characteristic of psychrophilic proteins to be stable at low temperatures when compared with other mesophilic chitinases [24,31] expressed in *P. pastoris*. Hong *et al*. [35] reported that high cultivation temperatures can induce the release of proteases from dead cells and may also affect the protein folding process. SDS-PAGE and western blotting analysis of the crude supernatants at the inducible period from 24 to 120 h showed two clearly visible protein bands with a molecular mass of approximately 39 and 50 kDa, respectively (Figure 4a and 4b). This study shows that *P. pastoris *is the suitable host for the production of cold-adapted protein such as CHI II. Similar finding for the high yield production of recombinant protein in methylotrophic yeast *P. pastoris *was obtained for other cold-adapted chitinase such as in the case of endochitinase from *Sanguibacter antarcticus *[36].

The recombinant chitinase CHI II protein (encoding CHI II mature protein) had an apparent molecular mass of about 39 kDa, less than the theoretically calculated molecular mass of 40.86 kDa. Another apparent band of about 50 kDa is higher than the calculated molecular mass and was predicted to be post-translationally modified and a glycosylated form of CHI II in *P. pastoris*. A GelCode Glycoprotein Staining kit was used to confirm the glycosylated moiety of the expressed chitinase in the polyacrylamide gel. As presented in Figure 4c, the 50 kDa band was positively stained, appearing as a magenta band with light pink background, whereas the 39 kDa band was not, indicating that the 50 kDa band was glycosylated. One putative N-glycosylation site (Asn-Xaa-Thr/ser) was found in the mature protein when the amino acid sequence were analysed using NetNGlyc 1.0 Server [27], while there was four potential O-glycosylation sites predicted using NetOGlyc 3.0 Server [37]. In eukaryotes, enhanced protein stability is often achieved by glycosylation resulting in protection by the attached sugar moieties. In *P. pastoris*, N-glycosylation is a common post-translational modification that enhances protein stability. Previous studies of chitinases from *Haemaphysalis longicornis *[38] and *Oryza sativa *L. [39] found larger proteins than the predicted molecular masses, implying that these chitinase proteins underwent post-translational modification.

### Enzymatic properties of purified CHI II

**Figure 5:**
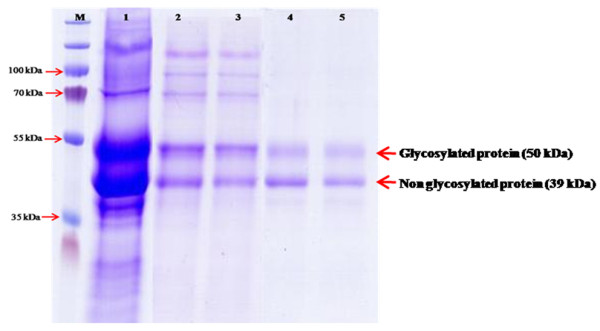
**Purification of recombinant chitinase (CHI II)**. Approximately 20 μl of sample was loaded into each well. M: protein molecular mass marker; Lane 1: crude concentrated enzyme; Lane 2 & 3: unbound fraction; Lane 3 & 4: elution fraction.

To examine the catalytic properties of CHI II produced by *P*. *pastoris*, the recombinant enzyme was purified to homogeneity using HisTrap ™ HP Columns from GE Healthcare. The purified recombinant chitinase CHI II was resolved as two bands by SDS-PAGE as shown in Figure 5.

**Figure 6:**
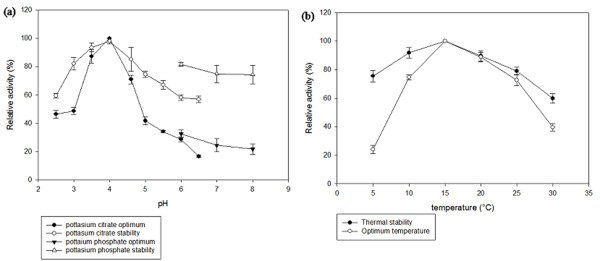
**Effect of pH (a) and temperature (b) on chitinase activity and stability**. The highest chitinase activity was set to 100%.

To determine the pH and temperature optima of the CHI II, the enzyme's activity was assayed at different pH levels and temperatures. The effect of pH on the chitinolytic activity was studied with a citrate-phosphate buffer (pH 2.5 to 6.5) and a potassium phosphate buffer (pH 6.0 to 8.0). Purified CHI II exhibited enzymatic activity over a pH range of 2.5 to 6.5 (measured at 15°C) and the activity of the purified chitinase was increased with increasing pH up to pH 4.0 in citrate-phosphate buffer. It was found that the optimal pH was 4.0 with more than 80% of the relative chitinase activity retained at the acidic condition of pH 3.5, as shown in Figure 6a. Alternatively, the optimum pH for chitinases produced by the psychrotolerant bacterium *Vibrio *sp. strain Fi:7 was pH 8.0 [25] while cold-adapted chitinase (ChiB) from a marine bacterium, *Alteromonas *sp. Strain O-7 is pH 6.0 [17]. At pH 4.5, the enzyme activity began to decline, resulting in a relative chitinase activity below 80%, while at pH 6.5, the relative activity was only about 10%. Even though the chitinase activity declined, purified chitinase was found to be stable. More than 80% of the maximum activity was retained at pH 3.0 to 3.5, but activity began to be lost after an incubation at a pH over 4.0. Extreme pH levels reduced the enzyme's ability to perform its catalytic activity. Most of the fungal chitinolytic enzymes have an optimum pH between 4.0 and 7.0 [40].

The effects of temperature on the recombinant chitinase's activity and stability were also determined. The optimum temperature for CHI II activity was 15°C (Figure 6b). The thermal stability of chitinase was measured by incubating an aliquot of the enzyme at different temperatures for 30 min and then assaying the residual activity under optimal pH and temperature conditions. Chitinase was stable at 15°C for 30 min and retained more than 90% of its initial activity when incubated at 10°C. Incubation at 20°C resulted in a 20% loss of the residual activity, while more than 70% of the maximal activity was retained when the enzyme was incubated at 5°C and 25°C. Moreover, the residual activity of the enzyme was 50% lower when incubated at 30°C. In general, cold-adapted enzymes display an apparent optimal activity shifted toward low temperatures and also heat lability [17]. It has been proposed that increased flexibility is the most important factor for the catalytic efficiency of cold-adapted enzymes at low temperatures [17], which is achieved through a combination of structural features [16]. Noticeably, the optimum temperature of CHI II (15°C) was much lower than those reported for the chitinase from *Moritella marina *(28°C) [7], cold-adapted chitinase B of *Alteromonas *sp. strain O-7 (30°C) [17] and chitinase A of *Vibrio *sp. strain Fi:7 (30°C) [25], yet the reasons for this finding remain unclear.

**Figure 7:**
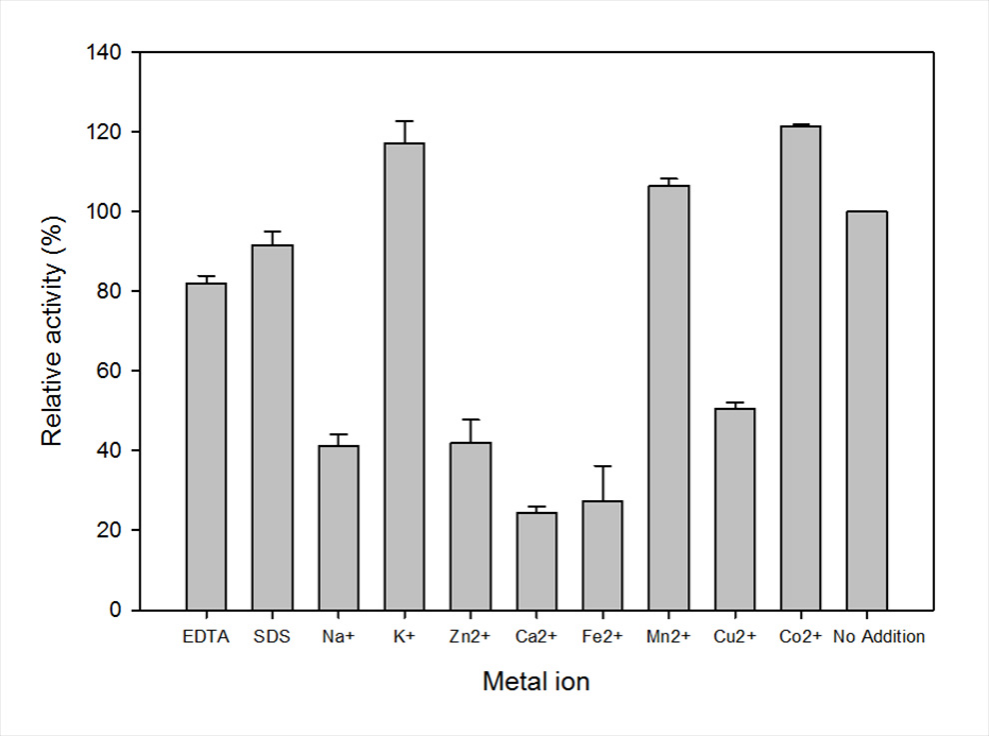
**Effect of metal ions and reagents on the recombinant CHI II chitinase activity**. No addition (with no metals added to the enzyme solutions) was used for 100% relative activity.

The catalytic activity of CHI II was strongly affected by the addition of Ca^2+ ^and Fe^2+ ^and was moderately inhibited by the other metal ions, such as Na^+^, Zn^2+ ^and Cu^2+^, which is comparable to the endochitinase from *Bacillus cereus *[41] (Figure 7). Chitinases from different fungi exhibit different responses to various metal ions [24]. It was reported that EDTA was an inhibitor of chitinase, e.g., it inhibited the chitinase from *Enterobacter *sp. NRG4 [9]. However, the addition of EDTA did not affect the catalytic activity of CHI II, suggesting that these chitinases may have different catalytic mechanisms. Differing from the *Trichoderma virens *UKM-1 endochitinase [42], which was completely inhibited by Mn^2+ ^and Co^2+^, CHI II was activated by the presence of both Mn^2+ ^and Co^2+^, which enhanced the activity about 10 to 20%. Moreover, K^+ ^also increased the activity of the recombinant CHI II to about 15%, while the CHI46 chitinase from *Chaetomium globosum *was inhibited by K^+ ^[31]. These results showed that chitinases could be activated or inhibited by certain metal ions. However, the chitinases from different species may be stimulated or inhibited by different ions.

Chitinases from different sources can use a variety of substrates. In this study, CHI II showed the highest activity toward colloidal chitin (0.690 U/ml) followed by swollen chitin (0.570 U/ml), carboxymethyl chitosan (0.465 U/ml), and glycol chitosan (0.278 U/ml). When a native chitinase from *G. antarctica *PI12 were tested against the same substrates, highest activity was exhibited with colloidal chitin (0.424 U/ml), followed by swollen chitin (0.340 U/ml), carboxymethyl chitosan (0.320 U/ml), and glycol chitosan (0.0615 U/ml). These observations show similar activity profile between the recombinant and native chitinase. The preference for hydrolysis of colloidal chitin over other substrates probably reflects that increased accessibility of colloidal chitin to the chitinase active site due to the removal of lipids and proteins from the crab shell chitin after acid hydrolysis treatment [43], while glycol chitosan is chemically different. The higher specificity to the colloidal chitin substrate was similar to that from *Trichoderma virens *[42]. However, its low hydrolytic activity against glycol chitosan was different from a previous study of chitinase from *Schizophyllum commune *[44].

Using the purified enzyme of CHI II, kinetic experiments were performed at a temperature of 15°C by varying the substrate concentration in a standard activity test from 2.0-10.0 mg ml^-1^. On the basis of the Lineweaver-Burk plot, the values of kinetic constants K_m_, V_max_, and subsequently k_cat_, and k_cat_/K_m _of CHI II towards different substrates (colloidal chitin, swollen chitin and carboxymethyl chitosan) were calculated as shown in Table 1. Using colloidal chitin as a substrate, a higher value of V_max_, which was 3.559 μmol μg^-1 ^h^-1^, was achieved compared to other substrates. The higher V_max _value indicates the higher efficiency of the enzyme and suggests that CHI II chitinase has a higher catalytic efficiency towards colloidal chitin as compared to the swollen chitin and carboxymethyl chitosan. The K_m _values of CHI II against different substrates were 27.918 mg ml^-1^, 13.83 mg ml^-1 ^and 26.79 mg ml^-1^, with colloidal chitin, swollen chitin, and carboxymethyl chitosan, respectively, which are comparatively higher than the other reports in literature [45,46]. Stefanidi *et al*. [7] suggested that enzymes produced by marine bacteria work at saturating concentrations of chitin and showed the high values of the K_m _constant. On the contrary, some cold-adapted enzymes have a lower K_m _than their thermostable homologues. For secreted enzymes from marine microorganisms, the requirement for a low K_m _may relate to the need to scavenge substrates that are at low concentrations in the environment [47].

**Table 1:** Kinetic parameters of CHI II on different chitin substrates

	V_max_ (μmole μg^-1 ^h-^1^)	K_m_ (mg ml^-1^)	K_cat_ s^-1^	k_cat_/K_m_ ml mg^-1 ^s^-1^
Colloidal chitin	3.559	27.918	0.915	0.0328
Swollen chitin	2.695	13.83	0.693	0.0501
Carboxymethyl chitosan	2.725	26.79	0.701	0.0262

Furthermore, the K_cat _value of CHI II was also found to be highest with colloidal chitin, which was 0.915 s^-1 ^and followed by carboxymethyl chitosan (0.701 s^-1^) and swollen chitin (0.693 s^-1^). The results support the V_max _value that indicated that CHI II had a lower turnover efficiency towards carboxymethyl chitosan and swollen chitin than towards colloidal chitin. However, the specificity constant k_cat_/K_m _is generally a better indication of the catalytic efficiency than k_cat _alone [48]. Comparable with the K_m _value, the k_cat_/K_m _values suggested that CHI II had a higher catalytic efficiency towards swollen chitin than colloidal chitin and carboxymethyl chitosan at low temperatures. A previous study found that a higher K_m _and k_cat _are also characteristics of lactate dehydrogenase (LDH-A4) enzymes from a cold-water fish where a higher K_m _results in a decrease in ΔG_ES_, with a concomitant decrease in the energy of activation required to form the transition state, thereby increasing the k_cat _[49]. Therefore, the strategy used to maintain sustainable activity at a permanently low temperature is to enhance the k_cat _and k_cat_/K_m _values instead of decreasing the K_m _[50].

## Conclusion

In this study, a chitinase from *G. antarctica *PI12 was isolated, purified and characterised. The protein displays an optimum catalytic activity at an apparently low temperature and pH. Due to its high versatility regarding its pH range, temperature range and substrate specificity towards chitin polymers, the *G. antarctica *PI12 family 18 chitinase seems to be a highly attractive enzyme for the production of chitooligosaccharides, and more generally for biotechnological applications such as for the biocontrol of microbial spoilage of refrigerated foods and use as a mycoparasite of phytopathogenic fungi in cold environments.

## Methods

### Microorganisms, plasmids, growth media, enzymes and reagents

The psychrophilic yeast, *G. antarctica *PI12 was obtained from School of Biosciences & Biotechnology, Universiti Kebangsaan Malaysia, Malaysia. *Escherichia coli *JM109 (Promega) was used as cloning host. The pPICZ*α*A vector (Invitrogen), which can propagate in both bacterial and yeast systems, was used for initial cloning in bacteria and subsequent expression in yeast. *P. pastoris *GS115 (Invitrogen) was used for heterologous protein expression. *G. antarctica *PI12 was grown on Yeast extract Peptone Dextrose (YPD) and chitinase induction medium (0.3% (w/v) yeast extract, 0.5% (w/v) peptone, 0.3% (w/v) NaCl and 3% (w/v) colloidal chitin), both containing 25 μg/ml ampicillin and 25 μg/ml kanamycin. The *G. antarctica *PI12 cells were incubated at 4°C for 7 to 8 days. *E. coli *JM109 was grown in Luria Bertani (LB) medium with 100 μg/ml ampicillin as a selectable antibiotic. Media and protocols used for *P. pastoris *are described in the *Pichia *expression manual (Invitrogen). Restriction enzymes were obtained from Promega and New England Biolabs (NEB), while all other chemicals were of analytical grade and were obtained from Sigma, Amresco, Fluka or Merck.

### Total RNA isolation and cDNA synthesis of the full-length chitinase gene

Total RNA was extracted from *G. antarctica *PI12 using a method as described by Sokolovsky *et al*. [51]. Briefly, *G. antarctica *PI12 was grown at 4°C for seven days in chitinase induction medium. RNA was purified and used immediately for cDNA synthesis or stored at -80°C. All primers used in PCR amplifications are listed in Table 2. The partial cDNA fragment of *G. antarctica *PI12 chitinase was amplified using primers LChi (F) and LChi (R) and an RT-PCR System (Promega), as recommended by the manufacturer. The primers were designed based on the Genome Sequencing Survey (GSS) Database of *G. antarctica *PI12 available at Malaysia Genome Institute (MGI). The resulting DNA fragment was used as a template for a subsequent RACE amplification to obtain the full-length CHI II sequence. 3' RACE was performed using primer 3-RC and a CapFishing™full-length cDNA Premix kit (Seegene) while 5' RACE was performed using primer 5-RC and a SMART™ RACE cDNA Amplification kit (Clontech), as recommended by the manufacturers. Using the sequence information from the RACE result, the full-length gene of chitinase was amplified using primer CHI-Rev and CHI-For via RT-PCR. DNA sequences amplified by PCR were confirmed by nucleotide sequencing (First BASE Laboratories).

**Table 2:** PCR primers used in this study

Primer name	Sequence (5'-3')	Orientation	Use
LChi (F)	GAGAATCGCAACTGACTT	Forward	RT-PCR
LChi (R)	GCAGGTGAATTCAGACGG	Reverse	RT-PCR
3-RC	AGGAAACCAGATCGGGATGT ACTGGCC	Forward	RACE amplification
5-RC	GTCGTCGCGCTCCAGGAGCCATAAAT	Reverse	RACE amplification
CHI-For	ATGAAGATCCCTCTCCTCTCCTCC	Forward	RT-PCR
CHI-Rev	CTACGCCTTGAACGTCCCCGCCAGT	Reverse	RT-PCR
CHI-*Not*I	TTTGCGGCCGCCTCACCCCTACCACTCCCTC	Forward	PCR
CHI-*Xba*I	TTTTCTAGAAACGCCTTGAACGTCC CCGCCAGT	Reverse	PCR

The location of *Not*I and *Xba*I are underlined

### Multiple sequence alignment and phylogenetic analysis

The phylogenetic relationship of CHI II was generated with 24 other deduced chitinases available from the NCBI databases as shows in Table 3. A phylogenetic tree was constructed by multiple sequence alignment using clustal X [52] and was generated using the Neighbour-Joining method (NJ) and bootstrap analysis. The phylogenetic tree was visualised using Treeview software. Confidence values for individual branches were assessed from 1000 bootstrap replicates of the original sequence data.

**Table 3:** Chitinases from different organisms used in the phylogenetic analysis

Taxanomy	Name/source	Abbreviation	Accession No
Bacteria;			
Proteobacteria	*Collimonas fungivorans*	C.fungivorans	ACF93784
Firmicutes	*Brevibacillus brevis *	B.brevis	YP_002771189
	*Paenibacillus *sp.	Paenibacil	ZP_04850994
	*Bacillus circulans*	B.circulans	AAF74782
	*Bacillus halodurans *	B.halodura	NP_241782
	*Kurthia zopfii*	K.zopfii	BAA09831
	*Clostridium papyrosolvens *	C.papyroso	ZP_03226994
	*Bacillus coahuilensis *	B. coahuile	ZP_03226994
	*Bacillus thuringiensis *	B. thuringi	ABQ65137
	*Bacillus mycoides*	B.mycoides	ZP_04167090
Bacteroidetes	*Rhodothermus marinus*	R.marinus	ZP_04424886
	*Spirosoma linguale*	S.linguale	ZP_04488661
Archaea;			
Euryarchaeota	*Thermococcus kodakarensis *	T.kodakare	YP_184178
	*Halogeometricum borinquense*	H.borinquense	ZP_04000472
Eukaryota;			
Metazoa;			
Arthropoda;			
Hexapoda;			
Insecta	*Aedes aegypti *	A.aegypti	XP_001656054
	*Tribolium castaneum*	T. castaneu	NP_001038094
	*Culex quinquefasciatus*	C. quinquefasciatus	XP_001867701
Chordata;			
Craniata;			
Vertebrata	*Mus musculus*	M.musculus	ABK78778
Fungi;			
Dikarya;			
Basidiomycota	*Amanita muscaria*	A.muscaria	CAC35202.1
	*Puccinia triticina*	P.triticin	AAP42832.1
	*Laccaria bicolor *	L.bicolor	XP_001886180
	*Coprinellus congregatus*	C.congregatus	CAQ51152
	*Cryptococcus neoformans *	C.neoforma	XP_572898
	*Taiwanofungus camphoratus*	T.camphora	ABB90389

### Construction of the chitinase expression plasmid and yeast transformants

The mature CHI II sequence was PCR amplified using primers CHI-*Not*I and CHI-*Xba*I and with the full-length cDNA as a template. The resulting DNA fragment (1100 bp) was digested with *Not*I and *Xba*I before being ligated into the corresponding sites of the pPICZαA vector and termed plasmid CHI II-pPICZαA. The recombinant enzyme was constructed such that the native signal peptide of the *G. antarctica *PI12 chitinase was replaced by the *Saccharomyces cerevisiae *α-factor signal peptide and was cloned in frame with the C-terminal tag. The ligation product was transformed into *E. coli *JM109. The correct sequence of the expression plasmid was confirmed by DNA sequencing.

Transformation of recombinant CHI II into *P. pastoris *GS115 was performed as recommended by the manufacturer (Invitrogen). Briefly, CHI II-pPICZαA was linearised using *Pme*I. The purified DNA product (2-5 μg) was transformed into competent *P. pastoris *GS115 cells via electroporation. Transformants were selected by plating onto YPD agar plates containing 100 μg/ml zeocin. The methanol metabolisation phenotype (Mut^+^) of *P. pastoris *recombinants was analysed by colony PCR using universal primers 5' AOX and 3' AOX. Colonies that produced a 1700 bp (plus the size of the parent plasmid) PCR product were selected and kept for subsequent analysis.

### Expression of recombinant CHI II in *P. pastoris *GS115

*P*. *pastoris *GS115 transformants were grown in 100 ml of fresh Buffered Complex Medium containing Glycerol termed as BMGY medium (1% (w/v) yeast extract, 2% (w/v) peptone, 100 mM potassium phosphate (pH 6.0), 1.34% YNB, 4 × 10^-5^% biotin and 1% (v/v) glycerol) in a 1000 ml baffled flask at 29°C and 250 rpm until the culture reached an A_600 _of 2-6 (approximately 18-20 hours). To induce CHI II production in *P*. *pastoris*, the cells were harvested and resuspended in Buffered Complex Medium containing Methanol or known as BMMY medium (1% (w/v) yeast extract, 2% (w/v) peptone, 100 mM potassium phosphate (pH 6.0), 1.34% YNB, 4 × 10^-5^% biotin and 0.5% (v/v) methanol) using 1/5 of the original culture volume (20 ml). Absolute methanol was added every 24 hours to a final concentration of 1% (v/v) to maintain induction. To analyse expression levels and the optimal time post-induction for harvest, the culture supernatant was collected at 1, 2, 3, 4 and 5 days. Expression of secreted proteins was analysed by SDS-PAGE.

### Purification of recombinant chitinase

All purification steps were performed at 4°C. The crude enzyme was concentrated by an Amicon concentrator 10,000 MWCO (MILIPORE) followed by purification using HisTrap™ HP Columns (GE Healthcare). The purified protein was collected and characterised further. The purified proteins were analysed by SDS-PAGE on a 10% gradient gel.

### Measurement of enzyme activity and protein determination

Chitinase activity was measured using 3, 5-dinitrosalicylic acid (DNS) as described by Miller *et al*. [53] but with some modifications. The reaction mixture contained 0.25 ml of 10% colloidal chitin in 0.2 M sodium acetate buffer (pH 4.0) and 0.25 ml enzyme solution. After an incubation at 15°C for 1 h, the reaction was terminated by boiling at 100°C for 5 min. The reaction mixture was centrifuged at 8,000 × g for 1 min. Next, 0.75 ml of DNS reagent was added to the aliquots of 0.25 ml reaction mixture that was then boiled at 100°C for 10 min. After cooling, the reducing sugars that were released as a result of the chitinase activity were measured at 540 nm using a UV spectrophotometer. One unit (U) of the chitinase activity is defined as the amount of enzyme that is required to release 1 μmol of N-acetyl-β-D-glucosamine per hour under the assay conditions. Protein content was measured according to the method of Bradford [54] using bovine serum albumin (BSA) as a protein standard. The reaction was measured at a wavelength of 595 nm.

### SDS-PAGE, western blot and glycosylation analysis of chitinase

Sodium dodecyl sulphate-polyacrylamide gel electrophoresis (SDS-PAGE) was conducted to analyse the recombinant protein expression by the method of Laemmli [55] and the gel was then stained with 1% Coomassie Brilliant Blue R-250. Western blotting was performed by colorimetric detection using a His-Tag monoclonal antibody, as recommended by the manufacturer (Novagen). Glycoprotein sugar moieties in the polyacrylamide gel were detected using a GelCode glycoprotein staining kit (Pierce Biotechnology) according to the instructions provided by the manufacturer.

### Characterisation of purified CHI II

The optimum pH for the purified CHI II was evaluated at 15°C over a pH range of 2.5 to 8.0, using appropriate buffers (100 mM), citrate-phosphate buffer (pH 2.5 to 6.5) and potassium phosphate buffer (pH 6.0 to 8.0), under CHI II chitinase assay procedures. The pH stability of the enzyme was investigated further at 15°C by pre-incubation of the enzyme solutions in the described buffer systems in the absence of substrate for 30 min. The reaction mixture was then subjected to the CHI II chitinase assay and a pH profile was produced with the enzyme activity at the optimum pH set to 100%.

The optimum temperature for purified CHI II activity was measured by incubating the purified enzyme for 30 min at temperatures ranging from 5°C to 30°C. The thermostability of CHI II was also investigated at temperatures of 5°C to 30°C after incubation of the enzyme solutions in the absence of substrate for 30 min. A temperature profile was produced with the enzyme activity at the optimum temperature set to 100%.

Metal ions are generally considered to be important factors affecting microbial enzyme activity. The reaction mixture consisted of purified enzyme in 100 mM citrate buffer (pH 4.0) containing 1 mM metal ions (K^+^, Cu^2+^, Mn^2+^, Fe^2+^, Co^2+^, Ca^2+^, Na^+ ^and Zn^2+^) and different reagents (such as 1 mM EDTA and 1% SDS). The effect of these metal ions was investigated using the CHI II chitinase assay system. The system without any additives was used as a control.

### Substrate specificity and kinetic parameters

The substrate specificity of CHI II was determined by measuring the enzyme activity after incubation in 100 mM citrate buffer containing 1% of each substrate (colloidal chitin, swollen chitin, glycol chitosan and carboxymethyl chitosan) at pH 4.0 and 15°C for 1 h. The amount of reducing sugars produced was estimated by using the DNS method as described above. The kinetic parameters (K_m_, V_max_, k_cat_, and k_cat_/K_m_) of the purified enzyme were studied. Different substrate (colloidal chitin, swollen chitin and carboxymethyl chitosan) concentrations were used, ranging from 2.0 to 10.0 mg ml^-1^. The reaction rate versus substrate concentration was plotted to determine whether the enzyme obeys Michaelis-Menten kinetics. The Michaelis-Menten constant (K_m_) and maximum velocity of substrate hydrolysis (V_max_) were determined from the Lineweaver-Burk plots.

## Competing interests

The authors declare that they have no competing interests.

## Authors' contributions

NMM, RMI developed the concept and design the study. ANMR performed experiments and wrote the manuscript. AR, AMAM, FDAB, RMI edited the manuscript. RMI gave technical support and conceptual advice. All authors have approved the final article to be submitted as manuscript.

## References

[B1] Bae E Phillips GN Structures and Analysis of Highly Homologous Psychrophilic, Mesophilic, and Thermophilic Adenylate Kinases Journal of Biological Chemistry 2004 279 28202 28208 10.1074/jbc.M401865200 15100224

[B2] Cavicchioli R Siddiqui KS Andrews D Sowers KR Low-temperature extremophiles and their applications Current Opinion in Biotechnology 2002 13 253 261 10.1016/s0958-1669(02)00317-8 12180102

[B3] Collins T Meuwis M-A Gerday C Feller G Activity, Stability and Flexibility in Glycosidases Adapted to Extreme Thermal Environments Journal of Molecular Biology 2003 328 419 428 10.1016/s0022-2836(03)00287-0 12691750

[B4] Joseph B Ramteke PW Thomas G Cold active microbial lipases: Some hot issues and recent developments Biotechnology Advances 2008 26 457 470 10.1016/j.biotechadv.2008.05.003 18571355

[B5] Feller G Lonhienne T Deroanne C Libioulle C Van Beeumen J Gerday C Purification, characterization, and nucleotide sequence of the thermolabile alpha-amylase from the antarctic psychrotroph *Alteromonas haloplanctis *A23 Journal of Biological Chemistry 1992 267 5217 5221 1544904

[B6] Lonhienne T Mavromatis K Vorgias CE Buchon L Gerday C Bouriotis V Cloning, Sequences, and Characterization of Two Chitinase Genes from the Antarctic *Arthrobacter *sp. Strain TAD20: Isolation and Partial Characterization of the Enzymes The Journal of Bacteriology 2001 183 1773 1779 10.1128/JB.183.5.1773-1779.2001 PMC95064 11160110

[B7] Stefanidi E Vorgias C Molecular analysis of the gene encoding a new chitinase from the marine psychrophilic bacterium *Moritella marina *and biochemical characterization of the recombinant enzyme Extremophiles 2008 12 541 552 10.1007/s00792-008-0155-9 18368288

[B8] Kubicek CP Mach RL Peterbauer CK Lorito M *Trichoderma*: From genes to biocontrol Journal of Plant Pathology 2001 83 11 23

[B9] Dahiya N Tewari R Tiwari RP Hoondal GS Chitinase from *Enterobacter *sp. NRG4: Its purification, characterization and reaction pattern Electronic Journal of Biotechnology 2005 8 134 145

[B10] Gooday GW Physiology of microbial degradation of chitin and chitosan Biodegradation 1990 1 177 190

[B11] Patil RS Ghormade V Deshpande MV Chitinolytic enzymes: an exploration Enzyme and Microbial Technology 2000 26 473 483 10.1016/s0141-0229(00)00134-4 10771049

[B12] Dahiya N Tewari R Hoondal G Biotechnological aspects of chitinolytic enzymes: a review Applied Microbiology and Biotechnology 2006 71 773 782 10.1007/s00253-005-0183-7 16249876

[B13] Xiao X Yin X Lin J Sun L You Z Wang P Wang F Chitinase genes in lake sediments of Ardley Island, Antarctica Applied and Environmental Microbiology 2005 71 7904 7909 10.1128/AEM.71.12.7904-7909.2005 PMC1317360 16332766

[B14] McCreath KJ Specht CA Robbins PW Molecular cloning and characterization of chitinase genes from *Candida albicans* Proceedings of the National Academy of Sciences of the United States of America 1995 92 2544 2548 10.1073/pnas.92.7.2544 PMC42254 7708682

[B15] Henrissat B Bairoch A New families in the classification of glycosyl hydrolases based on amino acid sequence similarities Biochemical Journal 1993 293 781 788 10.1042/bj2930781 PMC1134435 8352747

[B16] Tronelli D Maugini E Bossa F Pascarella S Structural adaptation to low temperatures - analysis of the subunit interface of oligomeric psychrophilic enzymes FEBS Journal 2007 274 4595 4608 10.1111/j.1742-4658.2007.05988.x 17697122

[B17] Orikoshi H Baba N Nakayama S Kashu H Miyamoto K Yasuda M Inamori Y Tsujibo H Molecular analysis of the gene encoding a novel cold-adapted chitinase (ChiB) from a marine bacterium, *Alteromonas *sp. Strain O-7 The Journal of Bacteriology 2003 185 1153 1160 10.1128/JB.185.4.1153-1160.2003 PMC142845 12562783

[B18] Nakamura T Ishikawa M Nakatani H Oda A Characterization of cold-responsive extracellular chitinase in bromegrass cell cultures and its relationship to antifreeze activity Plant Physiology 2008 147 391 401 10.1104/pp.106.081497 PMC2330313 18359848

[B19] Fenice M Selbmann L Di Giambattista R Federici F Chitinolytic activity at low temperature of an Antarctic strain (A3) of *Verticillium lecanii* Research in Microbiology 1998 149 289 300 10.1016/s0923-2508(98)80304-5 9766230

[B20] Papa R Rippa V Sannia G Marino G Duilio A Recombinant protein expression system in cold loving microorganisms Microbial Cell Factories 2006 5 1 2

[B21] Feller G Thiry M Arpigny JL Gerday C Cloning and expression in *Escherichia coli *of three lipase-encoding genes from the psychrotrophic antarctic strain *Moraxella *TA144 Gene 1991 102 111 115 10.1016/0378-1119(91)90548-p 1864500

[B22] Jahic M Wallberg F Bollok M Garcia P Enfors S-O Temperature limited fed-batch technique for control of proteolysis in *Pichia pastoris *bioreactor cultures Microbial Cell Factories 2003 2 6 10.1186/1475-2859-2-6 PMC166161 12871597

[B23] Gan Z Yang J Tao N yu z Zhang K-Q Cloning and expression analysis of a chitinase gene *Crchi1 *from the mycoparasite fungus *Clonostachys rosea *(syn. *Gliocladium roseum*) The Journal of Mirobiology 2007 45 422 430 17978802

[B24] Wang Y-J Yang Q Cloning and Expression of a Novel Chitinase *chi58 *from *Chaetomium cupreum *in *Pichia pastoris* Biochemical Genetics 2009 47 547 558 10.1007/s10528-009-9251-5 19507018

[B25] Bendt A Hüller H Kammel U Helmke E Schweder T Cloning, expression, and characterization of a chitinase gene from the Antarctic psychrotolerant bacterium *Vibrio *sp. strain Fi:7 Extremophiles 2001 5 119 126 10.1007/s007920100179 11354455

[B26] Dyrløv Bendtsen J Nielsen H von Heijne G Brunak S Improved Prediction of Signal Peptides: SignalP 3.0 Journal of Molecular Biology 2004 340 783 795 10.1016/j.jmb.2004.05.028 15223320

[B27] Gupta R Brunak S Prediction of glycosylation across the human proteome and the correlation to protein function Pacific Symposium on Biocomputing 2002 310 322 11928486

[B28] Hunter S Apweiler R Attwood TK Bairoch A Bateman A Binns D Bork P Das U Daugherty L Duquenne L InterPro: the integrative protein signature database Nucleic Acids Research 2009 37 D211 D215 10.1093/nar/gkn785 PMC2686546 18940856

[B29] Wang S-Y Wu S-J Thottappilly G Locy RD Singh NK Molecular cloning and structural analysis of the gene encoding *Bacillus cereus *exochitinase Chi36 Journal of Bioscience and Bioengineering 2001 92 59 66 10.1263/jbb.92.59 16233059

[B30] Limón MC Chacón MR Mejías R Delgado-Jarana J Rincón AM Codón AC Benítez T Increased antifungal and chitinase specific activities of *Trichoderma harzianum *CECT 2413 by addition of a cellulose binding domain Applied Microbiology and Biotechnology 2004 64 675 685 10.1007/s00253-003-1538-6 14740190

[B31] Liu ZH Yang Q Hu S Zhang JD Ma J Cloning and characterization of a novel chitinase gene (*chi46*) from *Chaetomium globosum *and identification of its biological activity Applied Microbiology and Biotechnology 2008 80 241 252 10.1007/s00253-008-1543-x 18563407

[B32] Gan Z Yang J Tao N Liang L Mi Q Li J Zhang K-Q Cloning of the gene *Lecanicillium psalliotae *chitinase *Lpchi1 *and identification of its potential role in the biocontrol of root-knot nematode *Meloidogyne incognita* Applied Microbiology and Biotechnology 2007 76 1309 1317 10.1007/s00253-007-1111-9 17665191

[B33] Kawase T Saito A Sato T Kanai R Fujii T Nikaidou N Miyashita K Watanabe T Distribution and phylogenetic analysis of family 19 chitinases in *Actinobacteria* Appl Environ Microbiol 2004 70 1135 1144 10.1128/AEM.70.2.1135-1144.2004 PMC348904 14766598

[B34] Mattanovich D Gasser B Hohenblum H Sauer M Stress in recombinant protein producing yeasts Journal of Biotechnology 2004 113 121 135 10.1016/j.jbiotec.2004.04.035 15380652

[B35] Hong F Meinander NQ Jönsson LJ Fermentation strategies for improved heterologous expression of laccase in *Pichia pastoris* Biotechnology and Bioengineering 2002 79 438 449 10.1002/bit.10297 12115407

[B36] Lee SG Koh HY Han SJ Park H Na DC Kim I-C Lee HK Yim JH Expression of recombinant endochitinase from the Antarctic bacterium, *Sanguibacter antarcticus *KOPRI 21702 in *Pichia pastoris *by codon optimization Protein Expression and Purification 2010 71 108 114 10.1016/j.pep.2010.01.017 20100576

[B37] Julenius K MÃ¸lgaard A Gupta R Brunak S Prediction, conservation analysis, and structural characterization of mammalian mucin-type O-glycosylation sites Glycobiology 2005 15 153 164 10.1093/glycob/cwh151 15385431

[B38] You M Xuan X Tsuji N Kamio T Taylor D Suzuki N Fujisaki K Identification and molecular characterization of a chitinase from the hard tick *Haemaphysalis longicornis* Journal of Biological Chemistry 2003 278 8556 8563 10.1074/jbc.M206831200 12502707

[B39] Park S-M Kim D-H Truong NH Itoh Y Heterologous expression and characterization of class III chitinases from rice (*Oryza sativa *L.) Enzyme and Microbial Technology 2002 30 697 702

[B40] El-Katatny MH Gudelj M Robra KH Elnaghy MA Gubitz GM Characterization of a chitinase and an endo-beta-1,3-glucanase from *Trichoderma harzianum *Rifai T24 involved in control of the phytopathogen *Sclerotium rolfsii* Applied Microbiology and Biotechnology 2001 56 137 143 10.1007/s002530100646 11499921

[B41] Chen WM Chen GH Chen CS Jiang ST Cloning, expression and purification of *Bacillus cereus *endochitinase in the *Escherichia coli *AD494(DE3)pLysS Expression System Bioscience, Biotechnology and Biochemistry 2009 73 1172 1174 10.1271/bbb.80618 19420688

[B42] Alias N Mahadi N Murad A Bakar F Mahmood N Illias R Expression and characterization of *Trichoderma virens *UKM-1 endochitinase in *Escherichia coli* World Journal of Microbiology and Biotechnology 2009 25 561 572

[B43] Sandhya C Adapa LK Nampoothiri KM Binod P Szakacs G Pandey A Extracellular chitinase production by *Trichoderma harzianum *in submerged fermentation Journal of Basic Microbiology 2004 44 49 58 10.1002/jobm.200310284 14768028

[B44] Yano S Rattanakit N Wakayama M Tachiki T A chitinase indispensable for formation of protoplast of *Schizophyllum commune *in basidiomycete-lytic enzyme preparation produced by *Bacillus circulans *KA-304 Bioscience, Biotechnology and Biochemistry 2004 68 1299 1305 10.1271/bbb.68.1299 15215595

[B45] Bhushan B Hoondal GS Isolation, purification and properties of a thermostable chitinase from an alkalophilic *Bacillus *sp. BG-11 Biotechnology Letters 1998 20 157 159

[B46] Woo C-J Park H-D An extracellular *Bacillus *sp. chitinase for the production of chitotriose as a major chitinolytic product Biotechnology Letters 2003 25 409 412 10.1023/a:1022493226727 12882563

[B47] Siddiqui KS Cavicchioli R Cold-adapted enzymes Annual Review of Biochemistry 2006 75 403 433 10.1146/annurev.biochem.75.103004.142723 16756497

[B48] Feller G Molecular adaptations to cold in psychrophilic enzymes Cellular and Molecular Life Sciences 2003 60 648 662 10.1007/s00018-003-2155-3 PMC11138853 12785714

[B49] Fields PA Somero GN Hot spots in cold adaptation: Localized increases in conformational flexibility in lactate dehydrogenase A4 orthologs of Antarctic notothenioid fishes Proceedings of the National Academy of Sciences 1998 95 11476 11481 10.1073/pnas.95.19.11476 PMC21668 9736762

[B50] D'Amico S Gerday C Feller G Structural determinants of cold adaptation and stability in a psychrophilic α-amylase Biologia (Bratislava) 2002 57 213 219

[B51] Sokolovsky VY Kaldenhoff R Ricci M Russo VEA Fast and reliable mini-prep RNA extraction from *Neurospora crassa* Fungal Genetics Newsletters 1990 37 39 40

[B52] Thompson JD Gibson TJ Higgins DG Multiple Sequence Alignment Using ClustalW and ClustalX Current Protocols in Bioinformatics 2002 John Wiley & Sons, Inc 10.1002/0471250953.bi0203s00 18792934

[B53] Miller GL Use of dinitrosalicylic acid reagent for determination of reducing sugar Analytical Chemistry 1959 31 426 428

[B54] Bradford MM A rapid and sensitive method for the quantitation of microgram quantities of protein utilizing the principle of protein-dye binding Analytical Biochemistry 1976 72 248 254 10.1016/0003-2697(76)90527-3 942051

[B55] Laemmli UK Cleavage of structural proteins during the assembly of the head of bacteriophage T4 Nature 1970 227 680 685 10.1038/227680a0 5432063

[B56] Kuranda MJ Robbins PW Chitinase is required for cell separation during growth of *Saccharomyces cerevisiae* Journal of Biological Chemistry 1991 266 19758 19767 1918080

[B57] Fitches E Wilkinson H Bell H Bown DP Gatehouse JA Edwards JP Cloning, expression and functional characterisation of chitinase from larvae of tomato moth (*Lacanobia oleracea*): a demonstration of the insecticidal activity of insect chitinase Insect Biochemistry and Molecular Biology 2004 34 1037 1050 10.1016/j.ibmb.2004.06.012 15475298

[B58] Watanabe T Oyanagi W Suzuki K Ohnishi K Tanaka H Structure of the gene encoding chitinase D of *Bacillus circulans *WL-12 and possible homology of the enzyme to other prokaryotic chitinases and class III plant chitinases J Bacteriol 1992 174 408 414 10.1128/jb.174.2.408-414.1992 PMC205731 1729234

